# Response patterns of routinely measured inflammatory and coagulatory parameters in sepsis

**DOI:** 10.7717/peerj.7147

**Published:** 2019-06-21

**Authors:** Mirjam Bachler, Tobias Hell, Lukas Schausberger, Christine Schlömmer, Volker Schäfer, Marlies Liebensteiner, Katharina Schäffler, Bettina Schenk, Dietmar Fries, Petra Innerhofer, Christian Niederwanger

**Affiliations:** 1 University for Health Sciences, Medical Informatics and Technology, Institute for Sports Medicine, Alpine Medicine and Health Tourism, Hall, Austria; 2 Department of Mathematics, Faculty of Mathematics, Computer Science and Physics, University of Innsbruck, Innsbruck, Austria; 3 Department of General and Surgical Critical Care Medicine, Medical University of Innsbruck, Innsbruck, Austria; 4 Department of Anaesthesiology and Critical Care Medicine, Medical University of Innsbruck, Innsbruck, Austria; 5 Department of Pediatrics, Pediatrics I, Medical University of Innsbruck, Innsbruck, Austria

**Keywords:** Fibrinogen, C-reactive protein, Inflammation, Coagulation, Platelets, Sepsis, Survival, Liver failure, Bilirubin, Mortality

## Abstract

**Background:**

Sepsis is characterized by a pro-inflammatory and pro-coagulatory shift which can induce life-threatening complications. Close monitoring and risk stratification of sepsis patients is crucial for proper treatment and consequently patient outcome. Therefore, this study focuses on the response patterns of inflammatory and coagulatory parameters used in clinical routines to estimate the course of sepsis.

**Methods:**

A total of 1,110 patients diagnosed with sepsis were retrospectively analyzed to identify response patterns for risk stratification of routine parameters measured at the peak level of C-reactive protein. Cluster analysis was used and the differences in the patient characteristics and 28-day survival were assessed. Cox proportional hazards regression model for survival stratified by the clusters was performed.

**Results:**

The analyses revealed the parameters to have five distinct response patterns. These clusters reflect the etiology as well as the course of sepsis associated with different mortalities. Here, impairment of the liver plays a crucial role in the ability to appropriately respond to sepsis. Of the routinely measured parameters, C-reactive protein and antithrombin seem to be unspecific for stratification of septic patients. Adjusted for the individual clusters, survival was associated with an increase in fibrinogen (*p* = 0.0042), platelets (*p* = 0.0003) and PT (*p* = 0.001) as well as a decrease in leukocytes (*p* = 0.034).

**Conclusions:**

This study reveals that patients have distinct response patterns of inflammatory and coagulatory parameters depending on disease etiology. These patterns are associated with different mortalities although the patients have similar levels of C-reactive protein. Independently of the type of response, good coagulatory capacity seems to be crucial for patient survival.

## Background

An increasing number of patients are being hospitalized for sepsis, although the overall mortality rate among patients with sepsis is declining (Martin et al., 2003; Angus et al., 2001; Kumar et al., 2011). Nevertheless, an increase in severe sepsis with organ failure can be observed (Kumar et al., 2011; Vincent et al., 2018). Therefore, close monitoring and risk stratification of sepsis patients is crucial for proper treatment and consequently patient outcome.

Sepsis is characterized by a proinflammatory and procoagulatory shift that can induce life-threatening complications in response to dangerous and pathogen-associated molecular patterns, whereby both systems interact in a complex way (Levi & Poll, 2015). When immune response and coagulation become exaggerated during sepsis, tissue and organ damage occur (Singer et al., 2016). Consequently, hemostatic disorders contribute significantly to organ dysfunction and the high lethality of sepsis (Semeraro et al., 2012).

One key factor in coagulation is fibrinogen, which also has many immunomodulating properties (Berends et al., 2014; Pahlman et al., 2013). It is a player of the direct host defense in that it builds a physical barrier against invading pathogens when forming fibrin fibers (Berends et al., 2014). Additionally, fibrinogen and its cleavage products have antimicrobial properties against certain bacteria (Pahlman et al., 2013), creating a new link between the innate immune system and blood coagulation.

Platelet count and antithrombin levels change according to the course of sepsis (Lauterbach et al., 2006; Semeraro et al., 2018). Antithrombin has anti-inflammatory properties, while platelets play a crucial role in inflammatory response in addition to their role in coagulation and fibrinolysis (Oelschlager et al., 2002; Gros et al., 2015). Only in extreme concentrations are leukocytes associated with the progression of sepsis, where the leukocyte count is itself part of the definition of sepsis (Levy et al., 2003a; Bone et al., 1992; Levy et al., 2003b).

A direct association between sepsis and elevated C-reactive protein levels has been described in several studies (Schentag et al., 1984; Maury, 1989; Povoa et al., 1998; Yentis, Soni & Sheldon, 1995; Smith et al., 1995; Presterl et al., 1997). This acute phase protein is suitable as a daily measurement to monitor the course of sepsis and can be regarded as an indicator of successful treatment (Yentis, Soni & Sheldon, 1995).

However, our understanding is limited as to how the various components of the inflammatory and coagulatory systems interact in different clinical situations and underlying diseases although activation of inflammatory and coagulation pathways is important in the pathogenesis of sepsis. The clinical picture of sepsis is very heterogeneous and ranges from relatively minor systemic damage to septic shock with multiple-organ failure (Levy et al., 2003a; Goldstein, Giroir & Randolph, 2005). The patient clientele also differs greatly depending on constitution and underlying diseases. The aim of this study was to evaluate routinely measured inflammation and coagulation parameters for their predictive power in the course of sepsis.

## Methods

This is a retrospective analysis of 1,110 septic patients at the medical, post-surgical and trauma Intensive Care Units (ICU) of Innsbruck Medical University Hospital, Austria. The data set contains clinical data and routine laboratory parameters.

### Inclusion of patients

Patients with the diagnosis of sepsis or systemic infection, who were treated at the medical, post-surgical and trauma ICUs between November 2000 and July 2013 with suspected or proven infection were respectively screened for study inclusion.

Patients meeting the sepsis criteria according to the American College of Chest Physicians/Society of Critical Care Medicine (ACCP/SCCM) international sepsis definitions based on a proven or strongly suspected infection with at least two of the SIRS criteria were included in the study (Bone et al., 1992; Levy et al., 2003b). No exclusion criteria were defined. The study was approved by the institutional review board of the Medical University of Innsbruck (AN2013-0043).

### Data collection

Demographic variables such as age, sex and the diagnosed underlying disease were recorded. The C-reactive protein (CRP) level during the septic episode was used to harmonize the progression of sepsis between the patients. We chose peak of C-reactive protein since this parameter reflects the inflammatory process and is widely used in clinical routine for the surveillance of inflammatory diseases. Many studies have described an interrelation between an elevated C-reactive protein level and sepsis (Schentag et al., 1984; Maury, 1989; Povoa et al., 1998; Presterl et al., 1997).

Inflammatory and coagulatory routine parameters such as fibrinogen, platelets, antithrombin, prothrombin time (PT), activated partial thromboplastin time (aPTT), bilirubin and leukocytes were collected on the day of the peak C-reactive protein. In-hospital mortality was chosen as the main outcome parameter.

### Statistical analysis

A mathematician not involved in the study procedures and patient assessment (TH) was responsible for the statistical analyses using R, version 3.4.1. All statistical assessments were two-sided and a significance level of 5% was used.

Patients with only one missing parameter on the day of C-reactive protein peak were included in the analyses. Simple mean imputation was deemed sufficient to deal with missing data, i.e., not inducing significant bias. C-reactive protein, fibrinogen, platelets, antithrombin, aPTT, PT, bilirubin and leukocytes measured at the peak level of C-reactive protein entered model selection according to the Bayesian Information Criterion (BIC) for expectation maximization (EM) initialized by hierarchical clustering for parameterized Gaussian mixture models using the R package mclust (Fraley et al., 2012). Gaussian mixture models are used to identify subpopulations within an overall population. Such models do not require knowledge of the subpopulation to which a patient belongs, thus permitting the model to automatically learn the subpopulations. For more details we refer to Fraley et al. (2012).

We descriptively present the resulting clusters by depicting the constituting variables via boxplots. Patient characteristics and outcome variables are presented as n (%) for binary data and continuous data as medians (25th to 75th percentile).

Differences between clusters are assessed with Pearson’s Chi-squared test for binary variables and the Kruskal-Wallis test for continuous variables; pairwise differences with the Wilcoxon rank sum test and Fisher’s exact test. We provide Kaplan–Meier curves for survival during 28 days after peak C-reactive protein stratified by the clusters and assess differences in survival using the log-rank test. A Cox proportional hazards regression model for survival stratified by the clusters was fitted and corresponding hazard ratios are provided, with 95% confidence intervals.

## Results

A total of 1,110 patients met the sepsis criteria according to criteria defined by the American College of Chest Physicians/Society of Critical Care Medicine (ACCP/SCCM) international sepsis definitions based on a proven or strongly suspected infection with at least two of the SIRS criteria (Bone et al., 1992; Levy et al., 2003b).

**Figure 1:**
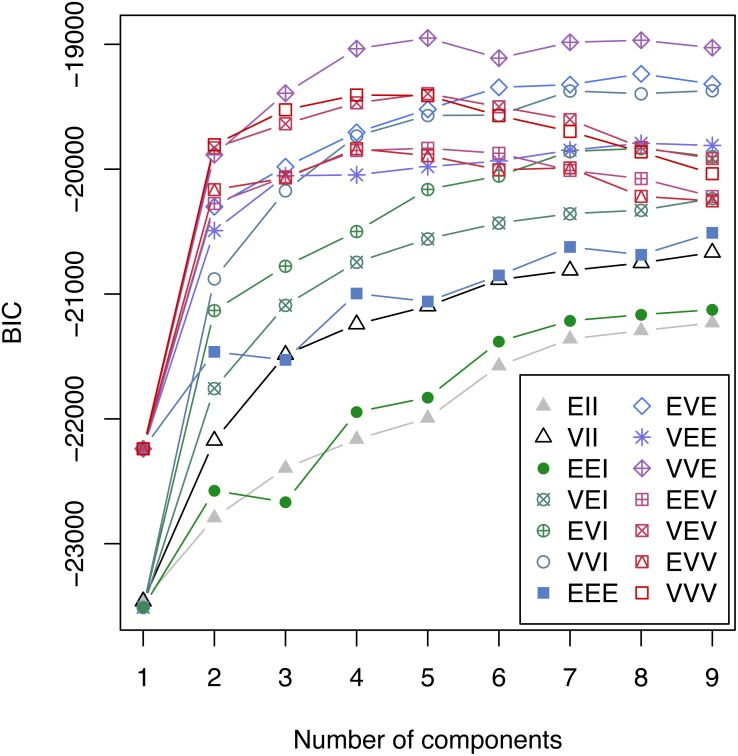
Bayesian Information Criterion (BIC) for expectation maximization (EM). BIC for all considered Gaussian mixture models with one to nine components. The highest BIC value is achieved by the VEE model with 5 components and consequently this clustering was selected.

79 patients were excluded from the final analysis because more than one of the analyzed parameters at the peak level of C-reactive protein was missing. The percentage of missing values in the remaining 1031 patients was 1.79%. C-reactive protein, fibrinogen, platelets, antithrombin, aPTT, PT, bilirubin and leukocytes at the peak level of C-reactive protein entered the model selection according to the Bayesian Information Criterion (BIC) for expectation maximization (EM) initialized by hierarchical clustering for parameterized Gaussian mixture models. A challenging task is to select the best model from a large class of models. A well established approach in the context of Gaussian mixture models uses the Bayesian Information Criterion (BIC) for expectation maximization. In Fig. 1 the BIC is given for all cluster solutions, depending on the number of clusters. The various cluster solutions are labeled with the abbreviations of the respective model. A list of all models is available in Fraley et al. (2012). The VVE (ellipsoidal, equal orientation) model with five clusters achieves the largest BIC value and was consequently selected.

### Patient population

In our patient population, men (65.5%) were more prevalent than women. Average age was 65 years and the majority of patients suffered from cardiovascular disease followed by complications of the digestive tract, as for example bowel perforation. The patients were severely ill and at ICU admission had a SAPS 3 score of 72 points and a SOFA score of 10 points. Of the patients 81.5% needed catecholamine therapy and 97.6% required mechanical ventilation.

### Cluster analysis

**Figure 2:**
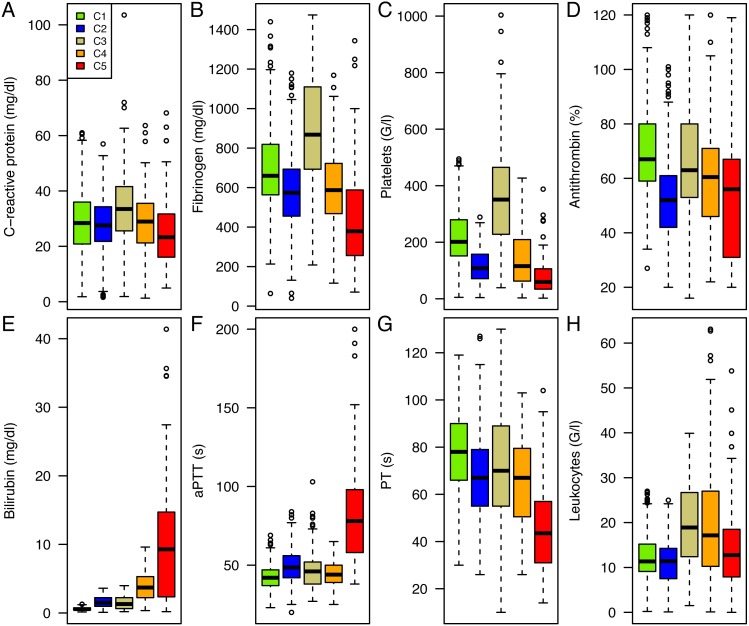
Cluster Analysis of routinely measured parameters at C-reactive protein peak. Boxplots of C-reactive protein (A), fibrinogen (B), platelets (C), antithrombin (D), bilirubin (E), aPTT (F), PT (G), and leukocytes (H). Parameters are depicted for cluster 1 (green), cluster 2 (blue), cluster 3 (golden), cluster 4 (orange), and cluster 5 (red) in (A to H).

The resulting five clusters (C1-C5) consisted of 336, 364, 105, 148 and 78 patients, respectively. Those clusters constituted profiles of patients presenting a similar pattern of routinely measured parameters at the peak level of C-reactive protein in sepsis (Fig. 2). All parameters differ significantly between the clusters (*p* < 0.0001).

**Figure 3:**
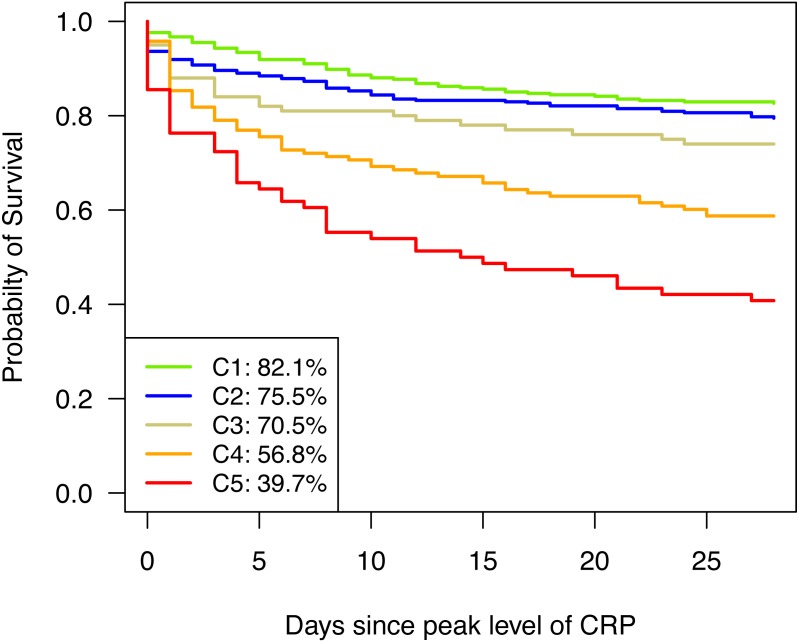
Kaplan–Meier curves for 28-day survival. Kaplan–Meier curves are depicted for cluster 1 (green), cluster 2 (blue), cluster 3 (golden), cluster 4 (orange), and cluster 5 (red).

The distinct patterns of parameters in the various clusters also reflected the type and severity of already existing diseases as SAPS 3 score increases from Cluster 1 to Cluster 5 (see Table 1). Correspondingly, 28-day mortality rose towards Cluster 5 (see Fig. 3) and differed significantly between clusters (*p* < 0.0001). Except for C1 and C2 as well as C2 and C3, which did not significantly differ from each other (*p* = 0.2576 and *p* = 0.2329, respectively), all other pairwise differences in 28-day survival were significant (see Table 2).

**Table 1:** Patient characteristics.^a^

		**CLUSTER**					
	**Total(*n* = 1, 031)**	**C1** (*n* = 336)	**C2** (*n* = 364)	**C3** (*n* = 105)	**C4** (*n* = 148)	**C5(*n* = 78)**	***P* value**^b^
Male gender	675 (65.5%)	219 (65.2%)	242 (66.5%)	67 (63.8%)	94 (63.5%)	53 (67.9%)	0.9426
Age (years)	65(53–75)	64(51–73)	69.5(56–77)	61(51–72)	64.5 (55.75–74)	61.5 (47.25–74.75)	0.0002
BMI (kg/m^2^)	25.39 (22.58–29.38)	25.95 (22.98–30.13)	24.67 (21.99–28.41)	26.12 (23.41–29.39)	25.71 (23.29–30.44)	24.22 (22.17–28.58)	0.026
**Diagnosed underlying disease**							
Cardiovascular	568 (55.1%)	167 (49.7%)	222 (61%)	45 (42.9%)	84 (56.8%)	50 (64.1%)	0.0012
Central nervous system	275 (26.7%)	111 (33%)	87 (23.9%)	31 (29.5%)	27 (18.2%)	19 (24.4%)	0.0061
Digestive tract	379 (36.8%)	115 (34.2%)	143 (39.3%)	48 (45.7%)	51 (34.5%)	22 (28.2%)	0.0799
Kidney	240 (23.3%)	70 (20.8%)	87 (23.9%)	22 (21%)	31 (20.9%)	30 (38.5%)	0.0171
Liver	137 (13.3%)	25 (7.4%)	52 (14.3%)	8 (7.6%)	26 (17.6%)	26 (33.3%)	<0.0001
Respiratory	351 (34%)	111 (33%)	133 (36.5%)	37 (35.2%)	44 (29.7%)	26 (33.3%)	0.6497
Skin	101 (9.8%)	38 (11.3%)	30 (8.2%)	15 (14.3%)	10 (6.8%)	8 (10.3%)	0.2124
Oncologic	191 (18.5%)	50 (14.9%)	69 (19%)	23 (21.9%)	33 (22.3%)	16 (20.5%)	0.2491
Need for catecholamines^c^	787 (81.5%)	213 (67.6%)	279 (80.2%)	79 (81.4%)	113 (86.3%)	62 (82.7%)	0.0136
Mechanical ventilation^d^	727 (97.6%)	225 (97.4%)	271 (97.7%)	78 (100%)	95 (96.9%)	58 (98.3%)	0.6671
SOFA score^e^	10 (8–12.25)	9 (7–11)	11 (9–12)	9 (7–11)	12 (10–14)	13 (11–16)	<0.0001
SAPS 3^e^ predicted mortality (%)	61 (40–79)	52 (32–72)	62 (46–78)	60 (41–78)	72 (50–83.25)	78 (48–89)	<0.0001
SAPS 3^e^ score	72 (62–84)	68 (58–79)	73 (65–83)	72 (62.5–83)	79 (67–88.25)	83 (66–95)	<0.0001

**Notes.**

a Binary data are presented as *n* (%), continuous data as medians (25th to 75th percentile).

b Assessed with Pearson’s chi-squared test for binary variables and the Kruskal–Wallis test for continuous variables.

c Not assessable for 65 patients.

d Not assessable for 286 patients.

e At admission, not assessable for 307 patients.

**Table 2:** Outcome.^a^

		**CLUSTER**					
	**Total(*n* = 1031)**	**C1** (*n* = 336)	**C2** (*n* = 364)	**C3** (*n* = 105)	**C4** (*n* = 148)	**C5(*n* = 78)**	***P* value**^b^
**Mortality**							
7-day	195 (18.9%)	32 (9.5%)	62 (17%)	24 (22.9%)	45 (30.4%)	32 (41%)	<0.0001
28-day	291 (28.2%)	60 (17.9%)	89 (24.5%)	31 (29.5%)	64 (43.2%)	47 (60.3%)	<0.0001
In-hospital	314 (30.5%)	70 (20.8%)	92 (25.3%)	35 (33.3%)	65 (43.9%)	52 (66.7%)	<0.0001
**Organ failure**							
Cardiovascular system	613 (59.5%)	169 (50.3%)	210 (57.7%)	66 (62.9%)	108 (73%)	60 (76.9%)	<0.0001
Central nervous system	341 (33.1%)	109 (32.4%)	131 (36%)	34 (32.4%)	39 (26.4%)	28 (35.9%)	0.3102
Intestine	207 (20.1%)	55 (16.4%)	75 (20.6%)	29 (27.6%)	29 (19.6%)	19 (24.4%)	0.1084
Kidney	584 (56.6%)	150 (44.6%)	223 (61.3%)	54 (51.4%)	96 (64.9%)	61 (78.2%)	<0.0001
Liver	291 (28.2%)	39 (11.6%)	111 (30.5%)	32 (30.5%)	59 (39.9%)	50 (64.1%)	<0.0001
Respiratory	591 (57.3%)	177 (52.7%)	209 (57.4%)	67 (63.8%)	90 (60.8%)	48 (61.5%)	0.1938
Multiple-organ dysfunction syndrome	596 (57.8%)	169 (50.3%)	222 (61%)	63 (60%)	89 (60.1%)	53 (67.9%)	0.0108
SOFA score^c^	10 (8–13)	8 (6–10.5)	11 (9–13)	9 (7–11)	12 (10–14)	14 (12–16)	<0.0001
Thromboembolic event	174 (16.9%)	45 (13.4%)	68 (18.7%)	23 (21.9%)	20 (13.5%)	18 (23.1%)	0.0617
Bleeding complication	284 (27.5%)	69 (20.5%)	117 (32.1%)	26 (24.8%)	43 (29.1%)	29 (37.2%)	0.0026

**Notes.**

a Binary data are presented as *n* (%), continuous data as medians (25th to 75th percentile).

b Assessed with Pearson’s chi-squared test for binary variables and the Kruskal–Wallis test for continuous variables.

c At peak of C-reactive protein, not assessable for 271 patients.

Patients in Cluster 1 were characterized by a moderate increase in inflammation and coagulation parameters in comparison to the other clusters describing a milder course of sepsis. The central nervous system was the main underlying disease, while the rate of other comorbidities was low as was the rate of mortality.

Typical for Cluster 2 were decreased levels of antithrombin as well as a relatively low platelet count. A further characteristic of this group was the high rate of renal disease and cardiovascular problems at ICU admission. This group suffered from an increased bleeding rate.

Cluster 3 showed a highly inflammatory course of sepsis with extremely elevated fibrinogen levels and a distinct probable reactive thrombocytosis. These patients frequently showed complications of the intestinal tract. Patients in C3 also exhibited a higher thrombosis rate although the difference was not statistically relevant.

Clusters 4 and 5 exhibited a state of decompensated coagulation and concomitant hyperbilirubinemia. Organ failure and the severity of dysfunction increased, resulting in elevated SOFA scores at the peak level of C-reactive protein. Liver failure frequently occurred in both clusters and was most pronounced in Cluster 5. 53.8% of the patients with a hepatic underlying disease suffered from pre-existing liver cirrhosis and of these patients, 71.4% developed acute-on-cirrhosis liver failure during the septic course (see File S1).

Sepsis severity was characterized by an increasing number of patients with multiple-organ failure towards Cluster 5. The disordered coagulation system resulted in an increased bleeding incidence in Clusters 2, 4 and 5. Especially in Cluster 5 80% of the patients with hepatic failure suffered from impaired liver synthesis and in most cases required vitamin K substitution. The thromboembolic events were most severe in the high inflammatory Cluster 3 and in Cluster 5, although the difference was not statistically significant. In Cluster 5 10.3% of the patients developed disseminated intravascular coagulation (DIC). The length of ICU stay did not significantly differ between clusters (*p* = 0.4085, Kruskal-Wallis rank sum test) and in median (IQR) lasted 14 (7–29) days.

### Timeline analysis

Since the patient data were collected over a protracted period from 2000 to 2013, the effect of time was investigated. The mortality rate was significantly higher in patients treated before 2005 than in patients treated later.

**Figure 4:**
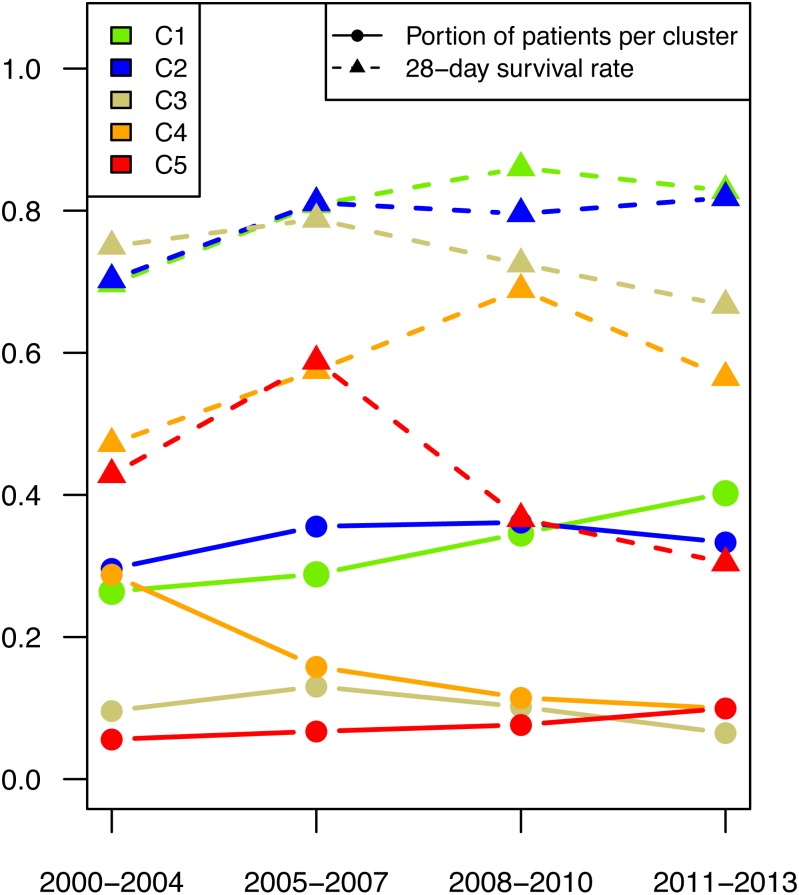
Patient number per cluster in time. The proportion of patients (dot) per time period and their respective 28-day mortality (triangle) are depicted for cluster 1 (green), cluster 2 (blue), cluster 3 (golden), cluster 4 (orange), and cluster 5 (red).

All types of clusters were present during the entire study duration, but there was a shift in patient numbers in the individual parameter patterns (see Fig. 4). The number of patients with the highest survival rate (shown in green in Fig. 4) increased over the years. Even when looking at the survival rate in this cluster, the 28-day survival rate was seen to have increased.

After a peak in the period 2004–2007 the number of patients in the high inflammatory response group (shown in gold in Fig. 4) decreased. The number of patients with mild liver failure declined significantly over the entire period, while the number in the group with severe liver failure increased.

### Coagulatory and inflammatory parameters associated with survival

**Figure 5:**
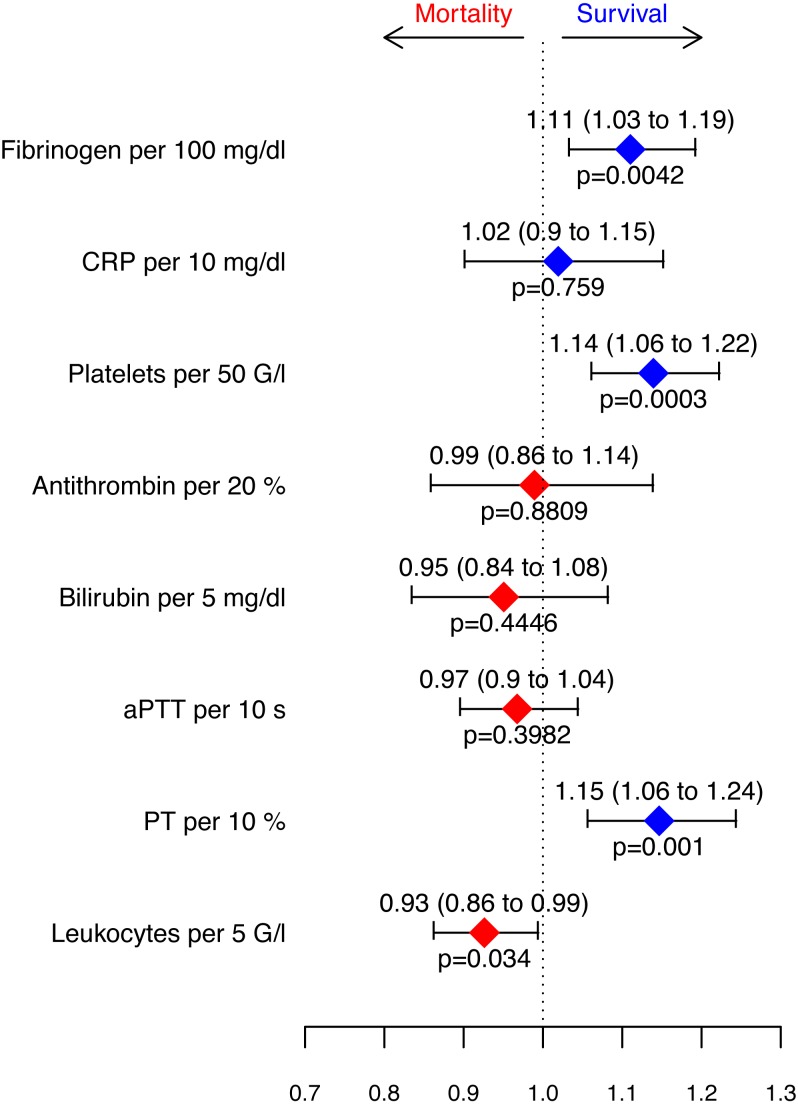
Hazard ratios retrieved from the Cox proportional hazards model for survival stratified by clusters. Depicted are increases in parameters.

A Cox proportional hazards regression model for survival stratified by clusters was fitted (Fig. 5) to evaluate the measured parameters with regard to their predictive value regarding the outcome. An increase in fibrinogen levels (100 mg/dl), number of platelets (50 G/l) and PT (10%) as well as a decrease in leukocyte count (5 G/l) was associated with improved 28-day survival.

## Discussion

In order to determine which routinely measured parameters of the immune system and the coagulation system are associated with good or poor outcomes with regard to various comorbidities during sepsis, a cluster analysis with subsequent Cox proportional hazards regression model for survival of these clusters was performed. Despite the different clinical features and the constellation of inflammatory and coagulative parameters, four significant parameters remained as independent predictors of survival in our patient population, regardless of the cluster assignment: fibrinogen, platelets, PT, and leukocytes.

### Cluster analysis

The cluster analyses resulted in five different response patterns that interestingly could be attributed to various underlying diseases.

Cluster 1 grouped patients with a low rate of comorbidities and a high probability of survival (Bassetti et al., 2017; Banta et al., 2012). Their response pattern was characterized by a mild inflammatory response and sufficient coagulatory capacity.

Patients in Cluster 2 frequently exhibited renal complications already at ICU admission, which was probably leading to protein loss or the need for hemodialysis with increased consumption of antithrombin (Lanquetot et al., 2008) and platelets (Stefanidis et al., 1996; Mulder et al., 2003).

Patients in Cluster 3, who frequently showed complications of the digestive tract, exhibited considerable hyper-inflammation and pronounced thrombocytosis. Thrombocytosis indicates the presence of a severe underlying disease (Tchebiner et al., 2011) and is associated with soft tissue infection, severe bacterial infection (Tchebiner et al., 2011; Fouzas et al., 2010) and a prothrombotic tendency (Rottenstreich et al., 2018; Ho, Yip & Duff, 2012). This could be true in our Cluster 3 patients since this group had the highest number of underlying digestive complications associated with a higher thrombosis rate although the difference was not statistically relevant.

Coagulation parameters were lowest in the patients in Clusters 4 and 5, probably as a sign of decompensation and multiple-organ failure, especially failure of the liver with subsequently elevated SOFA scores, resulting in impaired liver synthesis in 80% of all patients with liver failure. About half (53.8%) of the patients suffered from pre-existing liver cirrhosis and 71.4% experienced “acute-on-cirrhosis liver failure.” Cirrhosis seems to be a risk factor for developing impaired liver synthesis during sepsis, thus resulting in a higher rate of bleeding complications. Elevated bilirubin levels as surrogate marker for hepatic failure have already been shown to be associated with high mortality (Dizier et al., 2015). The extensive high share of developed cardiac organ failure (76.9%) and renal failure (78%) in Cluster 5 also indicates decompensation.

Hepatic failure leads to impaired synthesis performance resulting in desolate coagulation and declining levels of acute phase proteins in our patients. Prolonged aPTT and decreased PT indicate a hypocoagulant stage; especially low PT values seem to reflect the impaired synthetic capacity of the liver (Agarwal et al., 2012). In acute liver failure decreased levels of most coagulation factors are known (Agarwal et al., 2012). The antithrombin level decreases more quickly than other coagulation factors (Tischendorf et al., 2016), thus giving the illusion of a “rebalanced hemostasis” (Habib et al., 2014; Lisman & Porte, 2010). However, our septic patients with liver dysfunction showed a significantly increased bleeding rate as well as a trend towards a larger number of thromboembolic complications. These two conflicting complications seem to affect especially patients in Cluster 5, as they have impaired liver synthesis and are developing DIC (10%). Extremely increased monitoring of coagulation is essential for survival in this patient population.

The patients in these clusters are also characterized by lower numbers of platelets and leukocytes. This may be due to septic-induced immunosuppression (Schmoeckel et al., 2018; Venet & Monneret, 2018), in which patients are unable to make up for the increased consumption of platelets and leukocytes.

### Coagulatory and inflammatory parameters associated with survival

Despite the various clinical characteristics and constellation of inflammatory and coagulatory parameters, regardless of the cluster, four significant parameters remained as independent predictors for survival in our patient population, namely fibrinogen, platelets, PT and leukocytes.

An increase in fibrinogen levels per 100 mg/dl was associated with an 11% improvement in survival. The same observation was also made in a previous study (Ersoy et al., 2007). Moreover, sepsis animal models have confirmed that high fibrinogen levels are associated with better outcome (Journal of Cellular and Molecular Medicine Jr, Kinasewitz & Lupu, 2012; Fiusa et al., 2015; Luo et al., 2011; Sun et al., 2009; Mullarky et al., 2005). In our septic patient population low fibrinogen levels were associated with poor outcome, even if within the normal range.

Fibrinogen is not only responsible for the fibrin network during coagulation, but also plays a role in inflammation and the innate immune system (Davalos & Akassoglou, 2012). Both formation of the fibrin network and successive fibrinolysis exhibit an antimicrobial function by first capturing bacteria with subsequent lysis and so preventing further dissemination (Pahlman et al., 2013; Herwald, 2013; Loof et al., 2011; Wang et al., 2010). This can predominantly be observed in bacteria exhibiting fibrinogen binding sites, which is most common in gram-positive bacteria, such as Staphylococcus aureus, and also in some gram-negative bacteria, such as Moraxella catarrhalis (Pahlman et al., 2013) or Yersinia enterocolitica (Luo et al., 2011).

On the other hand, low fibrinogen levels may be due to consumption via fibrin deposition and subsequent disseminated intravascular coagulation (DIC) as well as multiple-organ dysfunction including liver damage (Semeraro et al., 2015; Parker, 2013). During liver failure the synthesis of fibrinogen is impaired (Nesseler et al., 2012) although in our patients with liver failure the fibrinogen levels were still mainly within or slightly above the normal range, but associated with poor outcome.

Thrombocytopenia during sepsis was associated with higher mortality, whereas the survival rate increased by 14% per 50 G/L of thrombocytes. A previous study revealed that the independent risk factors for thrombocytopenia during septic shock might be several comorbidities such as immunosuppression and liver cirrhosis, but are also assessed with severity of disease and site of infection (Thiery-Antier et al., 2016). Platelets are not only consumed by coagulation, but also by their immunological actions (Gros et al., 2015; Kim & Jenne, 2016; Speth et al., 2013).

Another parameter associated with a 15% improvement in survival when rising was the prothrombin time (PT) of 10%, namely independently of the cluster to which the patient was assigned. In some infectious diseases prothrombin time seems to be an important predictor for severe complications (Chang et al., 2019) and mortality in septic shock (Ishizuka, Tago & Kubota, 2014). Although the leukocyte count was described as a very weak prognostic marker for the survival of sepsis (Suberviola et al., 2012), we observed better survival in patients with fewer leukocytes.

Overall, coagulatory parameters appear to be well-suited for stratification of sepsis patients, particularly fibrinogen and platelets. Among other things, when both are elevated, they have a diagnostic value and may indicate a multi-pathogenic infection. When patients suffer from liver dysfunction, this is well reflected in prolonged aPTT, reduced PT and elevated bilirubin levels. In sepsis, a high fibrinogen level is associated with better survival while thrombocytopenia, leukocytosis, and low PT levels are associated with increased mortality. However, C-reactive protein and antithrombin appear to be unspecific for the stratification of septic patients.

### Timeline analysis

Patient data were collected over a protracted period from 2000 to 2013, and an effect of time on mortality is given. The mortality rate was significantly higher in patients treated before 2005 than in patients treated later. The decline in mortality rates among patients with sepsis was also observed in other studies (Martin et al., 2003; Angus et al., 2001; Kumar et al., 2011). In our study, all types of clusters were present during the entire study duration, but patient numbers shifted in the various parameter patterns.

The number of patients with the highest survival rate increased over the years, reflecting advanced therapy strategies. Interestingly, the number of patients with mild liver failure significantly decreased over the entire period, while the number in the group with severe liver failure increased. This is in accordance with other studies that showed that although the overall mortality from sepsis decreased, there was also a shift towards increased severity of sepsis and the number of organ failures (Kumar et al., 2011; Vincent et al., 2018).

This development of an increased survival rate is probably attributed to early and improved treatment of sepsis in order to not let sepsis become severe or the patients are able to survive until an extremely severe stage of disease when those patients are unresponsive to further therapy. This is supported by the fact that the cluster with severe liver failures shows the most distinct shift towards increasing mortality.

## Limitations

This study is limited in several aspects. The respective design of the study does not allow any assessment of causal relationships and merely gives associations. Nonetheless, the important role of a coagulatory situation, especially the high levels of fibrinogen and platelets related to improved outcome of sepsis, can be well explained by recently reported pathophysiological mechanisms. Furthermore, not all of the investigated parameters were available in every patient at the C-reactive protein peak. A prospective design would lower the number of missing values as well as offer the possibility to infer causality.

## Conclusion

The link between inflammation and coagulation plays a crucial role in critically ill patients with sepsis. Fibrinogen and platelets can serve as parameters for patient stratification during sepsis. When both are elevated, they also have a diagnostic value and may hint at a multi-pathogenic infection in the abdominal area. When patients suffer from liver impairment, this is well reflected in a prolonged aPTT, decreased PT and increased levels of bilirubin. C-reactive protein and antithrombin, however, seem to be unspecific for stratification of septic patients.

An increase in fibrinogen, platelets and PT is linked to survival independently of disease severity. Leukocytes are associated with increased survival in sepsis when declining.

In summary, good survival in sepsis is predicted better by a good coagulatory state than by the inflammatory situation reflected by C-reactive protein levels. The development of disease can be better followed by coagulation parameters than by inflammatory variables, except for leukocytes.

##  Supplemental Information

10.7717/peerj.7147/supp-1 File S1 Characteristics^*a*^ of patients in Cluster 5^*b*^

10.7717/peerj.7147/supp-2 File S2 Inflammatory and coagulatory parameters in Sepsis. Raw data applied for data analyses and preparation for all tables and figures.

## Abbreviations

ACCP/SCCM: American College of Chest Physicians/Society of Critical Care Medicine
aPTT: activated Partial Thromboplastin Time
BIC: Bayesian Information Criterion
C: Cluster
CRP: C-reactive protein
EM: Expectation Maximization
ICU: Intensive Care Unit
IQR: Interquartile Range
PT: Prothrombin Time (%)
SAPS: Simplified Acute Physiology Score
SIRS: Systemic Inflammatory Response Syndrome
SOFA: Sequential Organ Failure Assessment

## Additional Information and Declarations

### Data Availability

The following information was supplied regarding data availability: The raw data is available in the Supplemental File.
