# Supplementary material for: Response patterns of routinely measured inflammatory and coagulatory parameters in sepsis
Source: PeerJ. 2019 Jun 21;7:e7147. doi: 10.7717/peerj.7147 (PMC6590445; doi:10.7717/peerj.7147)
Supplement: File S1 [file peerj-07-7147-s001.docx]

Characteristics^a^ of patients in Cluster 5^b^

| **Underlying diseases** | | **Organ failure** | |
| --- | --- | --- | --- |
| **Cardiovascular diseases** | | | |
| Patients with cardiovascular underlying disease | 50 (64.1%) | Patients who developed cardiac organ failure | 60 (76.9%) |
| Aortic aneurysm / Dissection | 2 (4%) | Circulatory decompensation due to cardiac disease | 35 (58.3%) |
| Aortic dissection | 2 (4%) | Circulatory decompensation not due to cardiac disease | 21 (35.0%) |
| Cardiac arrhythmia | 4 (8%) | Ongoing underlying disease | 4 (6.7%) |
| Cardiomyopathy | 6 (12%) |  |  |
| Heart attack / Severe coronary heart disease | 11 (22%) |  |  |
| Heart valve defect | 5 (10%) |  |  |
| Myocarditis / Endocarditis | 5 (10%) |  |  |
| Circulatory decompensation not due to cardiac disease | 15 (30%) |  |  |
| **Renal diseases** | | | |
| Patients with renal underlying disease | 30 (38.5%) | Patients who developed renal failure | 61 (78.2%) |
| Chronic kidney failure | 14 (46.7%) | Acute kidney injury | 37 (60.7%) |
| Hepatorenal syndrome | 2 (6.7%) | Acute-on-chronic kidney injury | 13 (21.3%) |
| Kidney transplantation | 1 (3.3%) | Ongoing underlying disease | 11 (18%) |
| Nephrectomy | 1 (3.3%) | Need for renal replacement therapy (RRT) | 46 (75.4%) |
| Nephropathy | 1 (3.3%) |  |  |
| Acute kidney injury | 10 (33.3%) |  |  |
| Renal cell carcinoma | 1 (3.3%) |  |  |
| **Hepatic diseases** | | | |
| Patients with hepatic underlying disease | 26 (33.3%) | Patients who developed hepatic failure | 50 (64.1%) |
| Cholestatic hepatitis | 1 (3.8%) | Acute liver failure | 27 (54%) |
| Fatty liver | 3 (11.5%) | Acute-on-cirrhosis liver failure | 10 (20%) |
| Klatskin tumor | 2 (7.7%) | Liver transplantation failure | 1 (2%) |
| Liver abcess | 1 (3.8%) | Mild liver dysfunction | 10 (20%) |
| Liver cirrhosis | 14 (53.8%) | Oncologic liver failure | 2 (4%) |
| Liver transplantation failure | 1 (3.8%) | Impaired liver synthesis | 40 (80%) |
| Acute liver failure | 4 (15.4%) |  |  |

^a^ Binary data are presented as n (%)

^b^ Total number of patients in Cluster 5 is 78
